# The Relation of Rheumatic Diathesis to Common Throat Inflammations*Paper read before the West End Medical Society of New York City, October, 1896.

**Published:** 1897-02

**Authors:** James Edward Newcomb

**Affiliations:** Attending Laryngologist to Demilt Dispensary and to the Out-Patient Department of Roosevelt Hospital, New York City; Fellow American Laryngological Association


ATLANTA

Medical and Surgical Journal.

Vol. XIII. FEBRUARY, 1897. No. 12.

LUTHER B. GRANDY, M.D.,
MANAGING EDITOR.

M. B. HUTCHINS, M.D.,

BUSINESS MANAGER.

COLLABORATORS:
A. W. CALHOUN, M.D., LL.D., VIRGIL O. HARDON, M.D., FLOYD W. McRAE, M.D., DUNBAR ROY, M.D., AND HENRY R. SLACK, PH.M., M.D., LAGRANGE, GA.

### ORIGINAL COMMUNICATIONS.

### THE RELATION OF THE RHEUMATIC DIATHESIS TO THE COMMON THROAT INFLAMMATIONS.*

By JAMES EDWARD NEWCOMB, M.D.,

Attending Laryngologist to Demilt Dispensary and to the Out-Patient Department of Roosevelt Hospital, New York City; Fellow American Laryngological Association.

This topic is one of interest alike to the general and to the special practitioner, and as it has never been discussed before this society, it occurred to me that its consideration this evening might not be without profit to us. The time at my disposal forbids more than an outline of some of the many aspects the topic assumes.

A diathesis is defined as a congenital or acquired condition predisposing to some particular constitutional disease or to certain local manifestations of disease. In the case of rheumatism it is impossible to give, even at the present time, with all the advance in medical knowledge, a satisfactory definition. We do not yet

*Paper read before the West End Medical Society of New York City, October, 1896.

know the full range of its causative factors. In a general way it may be said to be due to the formation of some toxic material within the body or to inadequate elimination of some such material. Regarding the exact nature of the disease numerous theories are extant. Maclagan has referred its cause to the entrance into the body of some miasm from without; Recklinghausen and Klebs to a micro-organism entering the system and acting specifically on the fibro-serous structures as does small-pox on the skin. Heuter particularized this view, maintaining that the definite channels of entrance for the germ were the orifices of the ducts of the sweat-glands. Through the latter they were conveyed to the blood and caused an endocarditis. The joint lesions were the result of infectious emboli incident to the cardiac condition. Latham found the starting point of the malady in the hyper-oxidation of muscular tissue. The lactic acid theory of Prout is too well known to deserve more than mention. It is by no means universally accepted. The neurosal theory of Mitchell has had able advocates, and it is interesting to note that no less an authority than Jonathan Hutchinson regards rheumatism as a catarrhal neurosis, the exposure to cold and irritation of some area of skin or mucous membrane acting as the exciting factor. Still more recently, Riva has declared the pneumococcus of Fränkel to be the *materies morbi.* He regards not only endocarditis and pericarditis as rheumatic manifestations, but also pneumonia, pleurisy, cerebro-spinal meningitis, diplococcic septicemia with localizations, and every other infection, either local or general, produced by the above named organism.

Whatever elements of truth there may be in these various propositions, the exciting cause of rheumatism finds, by common consent, its origin in a perversion of the processes of assimilation and excretion, resulting in the formation of some unknown intermediate product or products of destructive metamorphosis. During the existence of such a condition, cold, the great exciting factor, acts directly upon either the vaso-motor or trophic nerves of the articulations, or else on the corresponding nerve centers through an influence on the centripetal nerve fibers.

In nearly every text-book on rheumatism the definition of the disease is usually followed by the statement that it is often associated with affections of the tonsils and pharynx. The relative frequency of this association is variously estimated by different authors. It is of some interest to note in passing that this matter has been seriously considered only during the last twenty-five years, though mentioned ten years before that era by Desnos, and in 1868 by Trouseau. In the latter year Lennox Browne boldly stated that the predisposing causes of rheumatism and tonsillitis were identical. In the last edition of his Treatise (1893) he practically reaffirms this view, holding that the dartrous or arthritic diathesis invariably exists in those patients subject to recurrent attacks of acute tonsillitis. In 1887 Haig Broun published notes of 345 cases of common throat inflammations, in 33 of which peri- and endo-cardial complications supervened without any antecedent rheumatic history; again in 1888 notes of 119 cases, 28 of them having rheumatic pains, 38 rheumatism and 10 rheumatic parents; that is, 64 per cent. of the whole number had some rheumatic tendency.

Fowler believes that careful inquiry will show that 80 per cent. of all cases of rheumatism have had previous sore throats.

Humphreys, in a group of some 240 cases of illness in 160 different patients, found 39 of acute or sub-acute rheumatism, 78 of tonsillitis and 138 of pharyngitis, while the balance closely resembled acute rheumatism in their signs and symptoms, including some cases of erythema and urticaria.

Knox has published a report of fifty cases of quinsy, in forty-five of which a distinct history of rheumatism was found, and in all of which the alkaline salicylates proved a specific in treatment.

Bean considers the rheumatic diathesis as the most important factor in the etiology of quinsy, and states as his personal experience that in fully 70 per cent. of his cases has there been a decided rheumatic history.

J. Solis-Cohen holds that tonsillitis may be rheumatic or non-rheumatic, and that for therapeutic purposes a distinction between these two forms is most important.

Now, on the other hand, Wm. Osler affirms that it is not in line with his experience to find any connection whatever between rheumatism and tonsillitis except that acute rheumatism is not infrequently preceded by inflammation of the tonsils. Mackenzie, Strumpell and Sajous, while casually admitting that a rheumatic diathesis may be the cause of a tonsillitis, broadly assign atmospheric influences as the more prominent feature in the cause of the disease. Max Thorner, in his etiology of quinsy, gives the disease a bacterial origin and discredits entirely the rheumatic theory, though he admits that the rheumatic virus may have an irritative action upon the throat in the production of what he fully describes as "rheumatic pharyngitis."

The foregoing quotations fairly represent the pros and cons of this mooted question.

Under such circumstances, where authorities of equal weight are arrayed against each other, one's convictions are naturally based upon his own experiences. For some years I have made it a routine to inquire into the family and antecedent personal history of all cases of acute sore throat with reference to rheumatism or any of its definite manifestations. Out of 586 patients questioned along this line, 154, or 26.2 per cent., presented either family or previous personal rheumatic trouble, while 432, or 73.8 per cent., were entirely free therefrom.

From this personal experience I am led to believe that the frequency of the rheumatic element in the common throat affections has been much overestimated. On the other hand, I well realize the unreliability of statistics collected in this manner, on account of two main reasons:

1. Patients are, as a rule, not accurate observers of their own symptoms, and are apt to respond too readily to leading questions so that we are able to draw out from them almost any statement we desire. The advocate of a favorite theory in medicine is always exposed to the danger of forgetting that he is really the judge of the case, and not the attorney either for the prosecution or defense.

2. Rheumatism is, as we all know, a composite disease. By no means are all of its features outlined in the portraiture of a single case. This is indeed true of all diseases, but it is notoriously manifest in rheumatism. Not only the joint lesions, but disturbances of the heart—itself called a "joint" by the great Louis—the nervous system, etc., all are the outposts where the advance guard of the enemy first gives battle. In regard to a chorea, Dyce Duckworth is quoted as saying that it is simply another variety of rheumatism in which the brain is affected instead of the joints, and he commends Sir Andrew Clark's definition of chorea as "Rheumatism of the Brain."

This fact is simply mentioned as suggestive of the care with which our questions should be propounded and the wide field they must cover in order to find out whether our patients have really had rheumatism or not.

Now, for our practical purpose, the great question is: How shall we decide whether a given case of throat trouble has an underlying rheumatic cause? Three phases of each case require consideration: The pathological, the clinical, and the therapeutical.

To take up the latter first, it is frequently found that the common throat affections yield to the alkalies and the salicylates, but here again are occasions for many deductions. These maladies are generally self-limited and stop in from 4 to 6 days without any treatment. In fact, much of our boasted success with any class of remedies, is confined to making the attack tolerable for the patient while not at all shortening its duration. If we begin our anti-rheumatic treatment on the fourth day, and the patient is well on the sixth, our remedies cannot, from that fact alone, be credited with any abortive power whatever. We should compare groups of parallel cases, contrasting those treated with those not treated; then again, the results in cases with a rheumatic history with results in those without such history. In my own cases, the therapeutical results have been the same in both classes of cases, So that, while not denying the truth of the main proposition of this paper, I think that a verdict of "cause and effect" is frequently rendered where one of "coincidence" would be nearer the truth.

As regards the clinical aspect, we may lay down the following rules to guide us in determining whether a given case is or is not of a rheumatic nature:

1. The exclusion of other manifest causative factors.
2. An antecedent personal rheumatic history.
3. A severity of local in comparison with general symptoms.

The pains in the back of the neck so often regarded as proof of rheumatic infection, are not necessarily so, for in many cases of sore throat, there is a mild septic infection from the absorption either of pus or of other toxic material, and to such infection the pains are frequently due.

The rheumatic cases generally begin with intense pain or deglutition, while the throat is but slightly congested. The saliva accumulates from the patient's unwillingness to swallow. The pain is frequently referred to the top of the esophagus. Respiration may also be painful and phonation excessively so. As the discomfort subsides, muscular soreness is felt in neck and loins. The joints stiffen up, but pain in them, as well as swelling and redness, is frequently wanting. The pain is apt to be more severe on the side of the body corresponding to the affected tonsil. It may shift from one tonsil to the other. Of course many cases of angina are followed by polyarthritis. In certain patients this follows so regularly that they know that when the sore throat begins they are in for a subsequent rheumatic outbreak.

Lastly, we have to inquire into the pathological identity, if any, between the lesions of the throat inflammations and rheumatism, and we may simplify our task by confining ourselves to tonsillitis and rheumatism.

It is regularly taught in all our text-books that the rheumatic poison affects the fibro-serous structures of the body, but the tonsil and its adjoining structures are not fibro-serous but fibro-mucous in character. Again, as age advances, the tonsils become less and less liable to inflammatory attacks, while the rheumatic tendency becomes more confirmed with passing years. " So far as quinsy is concerned, its course and culmination point so clearly to an infectious origin as to emphasize with every distinctness that it is a condition only accidentally, if at all, associated with a rheumatic diathesis."

Haig Broun again says: "Either rheumatism is a general disease which finds expression in the throat, OR else inflamed tonsils are receptacles for the rheumatic poison and media for its conductivity to the general circulation, OR still again specific germs find their way into the body under certain favoring conditions, then giving evidence of their presence by causing inflammation of the tonsils, fibrous and sero-fibrous structures;" and he adds, that in every case of tonsillitis we should examine the condition of the heart. It has been suggested that tonsillitis is really nothing but a mild attack of rheumatism. Most cases of the former are due to staphylococcus and streptococcus. Rheumatism shows in the first exudation the same morbific agents, and it is supposed that they have entered the body through the tonsils. An excess of the poison would overflow, so to speak, from the latter organs and if any point or points had been previously disturbed or possessed a weak nerve supply, the germs would pass out of the vessels and so produce an arthritis.

The foregoing seems to be a fair exposition of the question, as it is at present understood. It may be that subsequent researches into the nature of rheumatism will enable us to decide more definitely in regard to many points now mooted.

*118 West Sixty-ninth Street.*

